# The roles of long noncoding RNAs in myocardial pathophysiology

**DOI:** 10.1042/BSR20190966

**Published:** 2019-11-12

**Authors:** Cheng Chen, Yuting Tang, Hui Sun, Xiaofang Lin, Bimei Jiang

**Affiliations:** Department of Pathophysiology, Xiangya School of Medicine, Central South University, Changsha 410000, Hunan, P.R. China

**Keywords:** Biomarker, Cardiovascular disease, Long noncoding RNA, Myocardial pathophysiology

## Abstract

Long noncoding RNAs (lncRNAs), more than 200 nt in length, are functional molecules found in various species. These lncRNAs play a vital role in cell proliferation, differentiation, and degeneration and are also involved in pathophysiological processes of cancer and neurodegenerative, autoimmune, and cardiovascular diseases (CVDs). In recent years, emerging challenges for intervention studies on ischemic heart diseases have received much attention. LncRNAs have a key function in the alleviation of myocardial infarction (MI) injury and myocardial ischemia–reperfusion injury. During cardiac hypertrophy (CH) and fibrosis, cardiac cells undergo structural changes and become dysfunctional due to the effects of neurohormonal factors. LncRNAs may serve as important therapeutic targets that promote cardiac remodeling and then retard the development of heart failure (HF). In addition, studies on the roles and mechanisms of action of lncRNAs participating in cardiac pathophysiology via other factors have become the focus of research worldwide. Here, we review the current knowledge on various lncRNAs and their functions in cardiac biology, particularly concentrating on ischemic heart disease, CH, and cardiac fibrosis. We next discuss the predictive value of lncRNAs as diagnostic biomarkers of CVDs.

## Long noncoding RNA

Most of the human genome can be dynamically transcribed into noncoding RNAs (ncRNAs), whereas protein-coding genes account for approximately only 2–3% of the human genome [1]. NcRNAs can be roughly grouped into two classes based on size: small ncRNAs (<200 nt) and long ncRNAs (lncRNAs). LncRNAs, largely of unknown function, are broadly classified as transcripts longer than 200 nt with a limited coding potential. On the basis of their location relative to coding genes, these transcripts can be further categorized into five types: sense lncRNAs, antisense lncRNAs, bidirectional lncRNAs, intronic lncRNAs, and intergenic lncRNAs [2]. Over the past few years, as important regulators in the epigenetic, post-transcriptional, and translational coordination of gene expression in developmental and disease processes, lncRNAs have been proven to play a critical regulatory part in the organ development and pathophysiology [3]. Most studies have focused on the roles of lncRNA in development and differentiation, and many of the findings have implications in cancer biology. A growing body of evidence indicates that lncRNAs may participate in additional biological processes and that many lncRNAs correlate with diverse human diseases, among which, heart diseases are an emerging area of interest [4].

## The roles of lncRNAs in myocardial pathophysiology

### The expression profiling of lncRNAs in myocardial pathophysiology

Cardiovascular diseases (CVDs) are currently the main cause of morbidity and mortality. Cardiomyocyte injury as a fundamental pathophysiological feature of CVDs plays a pivotal part in the development of (and in) CVDs. It is implicated in almost all kinds of CVDs. Besides, various diseases of other systems of organs may provoke this process too. The strategies for prevention of such adverse events are clinically important. Lately, researchers worldwide screened lncRNAs for those differentially expressed in various heart diseases (Table 1).

**Table 1:** The expression profiles of lncRNAs in CVDs

Species	Models	Differentially expressed lncRNAs	Samples
*Homo sapiens*	Hypoxic condition	164: 8 were up-regulated by more than 1.5-fold and 18 were down-regulated by more than 1.5-fold (GSE50378)	HUVEC cells [5]
		119: 35 up and 30 down were taken as representative (GSE22282)	Mature dendritic cells [5]
Mouse	MI/RI	151: 64 up and 87 down (fold-change > 2.0)	Infarct region of heart [17]
Mouse	MIPostC corresponding to MI/RI	4140: 2292 up and 1848 down (absolute fold-change > 2.0) (vs. I/RI)	Heart tissues [18]
Mouse	The early stage of MI	642: 301 up and 341 down (FDR < 0.05, |log_2_FC| > 1)	Left ventricular tissues [19]
Mouse	MI	1173: 194 up and 979 down (FDR < 0.05, |log_2_FC| > 1)	Left ventricular tissues [19]
		133: 67 up and 66 down (UCSC-annotated)	Heart tissues [6]
		311: 86 up and 225 down (novel unannotated)	
Human	ICM	145: 108 up and 37 down	Myocardial samples [7]
Rat	AngiotensinII-induced autophagy	1249: 700 up and 549 down (≥two-fold change)	Neonatal cardiomyocytes [8]
Mouse	Cardiac fibrosis induced by MI	545: 263 up and 282 down (53 were up-regulated by 2.0-fold change and 37 down-regulated by 0.5-fold change)	Peri-infarct tissues of heart [9]
		198: 64 were up-regulated by	
Mouse	CH (isoproterenol)	2.0-fold change and 134 down-regulated by 0.5-fold change	Left ventricle tissues [10]
Rat	CH (TAC)	252: 80 up and 172 down (fold-change > 2.0)	Heart tissues [11]
Human	DCM	313: 139 up and 174 down (fold change > 2)	Heart tissues [12]
Rat	Ischemic HF (by ligating the LAD)	2600: 1197 up and 1403 down (fold-change > 2.0)	Heart tissues [13]
Human	HF	48: 13 up and 35 down (vs. unused RVs)	HF right ventricular (RV) tissues of patients [14]
		13: 10 up and 3 down (fold change ≥ |1|)	LV biopsies of patients [15]
		768: 550 up and 218 down (>two-fold change)	Plasma of patients [16]

Abbreviations: AMI, acute myocardial infarction; CH, cardiac hypertrophy; DCM, dilated cardiomyopathy; FC, fold-change; FDR, false discovery rate; HF, heart failure; ICM, ischemic cardiomyopathy; IPostC, ischemic postconditioning; LAD, left anterior descending coronary artery; MI, myocardial infarction; MI/RI, myocardial ischemia–reperfusion injury; TAC, transaortic constriction.

To explore the lncRNAs that take part in rat myocardial ischemia/reperfusion (MI/R) injury, Zheng et al. [5] have used Gene Expression Omnibus (GEO) to screen transcripts for differentially expressed genes and found 164 and 119 differentially expressed lncRNAs in datasets GSE50378 and GSE22282, respectively. Among 17584 reconstructed, multi-exonic transcripts sequenced from tissues in the border zone of mouse hearts in response to myocardial infarction (MI), Ounzain et al. [6] have identified 2509 multi-exonic lncRNAs. They reported 988 (67 up- and 66 down-regulated) University of California Santa Cruz-annotated lncRNAs and 1521 (86 up- and 225 down-regulated) novel unannotated lncRNAs [6]. Using RNA deep sequencing of ncRNAs in human cardiac tissues, Huang et al. [7] have found that 145 lncRNAs are differentially expressed in ischemic cardiomyopathy tissues compared with healthy control samples. Cardiomyocyte autophagy is implicated in various heart diseases. Gu et al. [8] have screened lncRNAs profiles in cardiomyocyte autophagy induced by angiotensin II (AngII) and identified 1249 differentially expressed lncRNAs using Genespring software, including 700 up- lncRNAs and 549 down-regulated lncRNAs. In addition, lncRNA microarray analyses performed in cardiac fibrosis [9], cardiac hypertrophy (CH) [10,11], dilated cardiomyopathy (DCM) [12], and heart failure (HF) [13–16] provide an experimental basis for future investigation of lncRNAs implicated in heart diseases.

### The roles of lncRNAs in MI

During MI, decreased or interrupted blood supply to the coronary artery can induce serious and lasting ischemic necrosis of myocardial cells. Cardiomyocyte apoptosis is the main characteristic of MI. Autophagy, an intracellular degradation process, has also been found to contribute to cardiomyocyte death in numerous heart diseases [17]. An increasing body of evidence suggests that lncRNAs play crucial roles in MI (Table 2).

**Table 2:** The roles of lncRNAs in MI

LncRNA	Models	Expression	Role
ZFAS1	AMI patients	Down	[21]
	AMI in rats	Up	Knockdown of ZFAS1 could relieve AMI-induced MI [22]
	MI in mice	Up	Knockdown of ZFAS1 could mitigate the ischemic contractile dysfunction of hearts [23]
HOTAIR	AMI in mice	Down	Limiting cardiomyocyte apoptosis [24]
	H9c2 cells under H_2_O_2_ treatment	Down	HOTAIR could relieve oxidative stress-induced H9c2 cells injury [25]
CAIF	Neonatal mouse cardiomyocytes under H_2_O_2_ treatment	Down	Inhibiting cardiac autophagy and ameliorating MI [26]
MALAT1	MI in mice and H9c2 cells treated with isoproterenol (ISO)	Up	Enhancing protective autophagy and decreased apoptosis of hearts in mice after MI [28]
	MI in mice and human ventricular myocyte AC16 cells under hypoxia	Up	Knockdown of MALAT1 could improve cell viability and inhibit cell apoptosis in hypoxia-induced cardiomyocytes [29]
GAS5	H9c2 cells under H_2_O_2_ treatment	Up	Knockdown of GAS5 could attenuate cell apoptosis in hypoxia-induced cardiomyocytes [32]
FAF	AMI in rats and neonatal rat primary cardiomyocytes under ischemia–hypoxia	Down	FAF could inhibit cell apoptosis ischemia–hypoxia induced cardiomyocytes [33]
TTTY15	Human cardiomyocyte primary cells (HCMs) under hypoxia condition	Up	Knockdown of TTTY15 could reduce hypoxia-induced cardiomyocytes injury [34]
ECRAR	MI in rats	-	Increasing rat post-natal CM proliferation after MI and improving post-MI cardiac function in adult rats [35]
CPR	MI in mice	-	Ablation of CPR could increase cardiomyocyte proliferation and improve cardiac function in mice after MI [36]
NR_045363	MI in mice	-	Increasing cardiomyocyte proliferation and improving cardiac function in mice after MI [38]
TUG1	Rat cardiomyocytes under hypoxia treatment	Up	Aggravating hypoxia-induced cell injury [40]
Meg3	Rat cardiomyocyte-derived H9c2 cells under hypoxia	Up	Knockdown of MEG3 could alleviate hypoxia-induced cell injury [42]

Abbreviations: AMI, acute MI; CAIF, cardiac autophagy inhibitory factor; CPR, cardiomyocyte proliferation regulator; ECRAR, endogenous cardiac regeneration-associated regulator; FAF, fibroblast growth factor 9-associated factor; GAS5, growth arrest-specific transcript 5; HOTAIR, HOX transcript antisense intergenic RNA; MALAT1, metastasis-associated lung adenocarcinoma transcript 1; TTTY15, testis-specific transcript Y-linked 15; ZFAS1, Zinc finger antisense 1.

#### Zinc finger antisense 1

Concentration of Zinc finger antisense 1 (ZFAS1), also known as heart-specific or heart-related lncRNA, has been found to be significantly lower in whole blood from patients with acute MI (AMI) (0.74  ±  0.07) compared with non-AMI patients and healthy volunteers by quantitative reverse-transcription PCR analysis [18]. Wu et al. [19] have examined the expression of ZFAS1 in rats with AMI and found that ZFAS1 is up-regulated in infracted myocardium zones within 48 h post-AMI and then its expression dramatically decreases at 1 and 2 weeks post-AMI as compared with no-infarction controls. ZFAS1 was also shown to perform some function in AMI via interaction with miR-150 in RNA pull-down assays. A knockdown of ZFAS1 or miR-150 overexpression attenuated decreased cell viability and enhanced expression of C-reactive protein (CRP) caused by hypoxia and reduced the extent of MI in rats at 1 week post-AMI [19]. Zhang et al. [20] have found that ZFAS1 expression is higher in the myocardium of AMI patients and in cultured neonatal mouse cardiomyocytes after exposure to a hypoxic environment for 12 h (a 2.6-fold increase). A knockdown of endogenous ZFAS1 attenuated ischemia-induced contractile dysfunction, whereas ZFAS1 overexpression impaired the contractility of cardiac muscles. Moreover, ZFAS1 induced intracellular Ca^2+^ overload via deleterious alteration of Ca^2+^ transit in cardiomyocytes. ZFAS1 was also found to have strong affinity for SERCA2a—a key protein involved in the maintenance of normal intracellular Ca^2+^ concentrations and cardiac contractile function—and to repress its expression as well as limit its activity [20].

#### HOX transcript antisense intergenic RNA

HOX transcript antisense intergenic RNA (HOTAIR), a modulator of *HOX* gene expression, has been reported to participate in cancer progression. Recent experiments were conducted to assess the expression and role of HOTAIR in AMI. Gao et al. [21] have found that the expression of HOTAIR is significantly decreased in the early phase of AMI and is the lowest 6–12 h after AMI compared with control groups. During the 3 days post-AMI, HOTAIR expression gradually returned to the levels seen in the control group. Similarly, expression of HOTAIR in the serum of mice with AMI and in cardiomyocytes exposed to hypoxia for 1, 6, and 24 h was shown to be down-regulated. Furthermore, HOTAIR overexpression induced by an adenovirus vector carrying the HOTAIR gene significantly reduced cardiomyocyte apoptosis under hypoxic conditions. Conversely, a knockdown of HOTAIR by AdshHOTAIR (adenoviral short hairpin HOTAIR) reversed the inhibitory effect. It was concluded that HOTAIR exerts its effect partly by interacting with miR-1 [21]. Li et al. [22] have discovered that HOTAIR is significantly down-regulated in H9c2 cells after hydrogen peroxide (H_2_O_2_) treatment, whereas overexpression of HOTAIR has cardioprotective effects against oxidative stress-induced injury. Additionally, HOTAIR acted as a sponge for miR-125 that modulates the HOTAIR–miR-125–MMP2 axis in H9c2 cells under oxidative stress. This finding may provide a novel target for AMI therapy [22].

#### Cardiac autophagy inhibitory factor

The expression of lncRNA known as cardiac autophagy inhibitory factor (CAIF) is decreased in cardiac tissue upon H_2_O_2_ treatment, and CAIF takes part in the regulation of p53 function in cardiomyocytes. CAIF may inhibit cardiac autophagy and ameliorate MI by directly binding to the p53 protein and blocking p53-mediated myocardin transcription. Myocardin regulates autophagy in cardiomyocytes during MI/R injury. Thus, CAIF, p53, and myocardin may represent a novel axis that regulates autophagy and exerts a therapeutic action in cardiomyocytes [23].

#### Metastasis-associated lung adenocarcinoma transcript 1

LncRNA metastasis-associated lung adenocarcinoma transcript 1 (MALAT1) was first identified as a prognostic parameter in non‐small-cell lung cancer [24]. Guo et al. [25] recently found that MALAT1 was highly expressed in the infarcted rat heart tissues after MI compared with adjacent non-infarcted regions. ‘MALAT1’ functioned as a protective factor in H9c2 cells treated with isoproterenol (ISO). Using dual luciferase reporter assay, researchers verified that MALAT1 directly targeted miR-558, which could aggravate ISO-induced cardiomyocytes apoptosis. In subsequent studies, it was demonstrated that Unc-51-like autophagy activating kinase 1 (ULK1), a downstream target of miR-558, involved in the modulation of miR-558 on the cell fate in cardiomyocytes treated with ISO. Through stimulating MALAT1–miR-558–ULK1 pathway, ISO-induced mitochondria-dependent apoptosis was improved in H9C2 cells. Furthermore, MALAT1-knockout mice were generated to confirm the protective actions of MALAT1 on the hearts under ISO treatment. Sun and Zhang [26] reported that MALAT1 could modulate the cellular processes of cardiomyocytes during MI by absorbing miR-200a-3p, thereby further up-regulating programmed cell death 4 (PDCD4), which was previously identified as a target of MALAT1 in MI. Thus, these studies reveal that MALAT1 may be a potent target for treatment of MI.

#### Growth arrest-specific transcript 5

LncRNA growth arrest-specific transcript 5 (GAS5) was proved to be a tumor suppressor involved in different types of cancers [27,28]. Du et al. [29] demonstrated the regulatory role of GAS5 in HF caused by MI. The data showed that loss of GAS5 expression reduced the cell death in hypoxia-treated H9c2 cells. Among the four HF-related miRNAs, GAS5 was validated as a sponge for miR-142-5p by bioinformatics analysis and RNA-binding protein immunoprecipitation (RIP) assay. Notably, GAS5 suppression activated the phosphoinositide 3-kinase (PI3K)–serine-threonine kinase (AKT) and mitogen-activated protein kinase 1 (MEK)–extracellular regulated protein kinase (ERK) signaling pathways possibly in an miR-142-5p-dependent manner, thereby resulting in alleviation of hypoxia-induced cardiomyocytes injury [29].

#### Fibroblast growth factor 9-associated factor

According to the high-throughput sequencing, GO and KEGG pathway analyses, researchers selected a highly conserved lncRNA fibroblast growth factor 9-associated factor (FAF) as a candidate lncRNA and investigated its regulatory role during AMI. The decreased FAF expression was observed both in neonatal rat primary cardiomyocytes under ischemia–hypoxia and cardiac tissues of AMI rats. The results displayed that FAF exerted significantly protective effects on ischemia–hypoxia cardiomyocytes and could regulate the expression of FGF9, which was previously verified as a protective factor in post-MI. FGF9 abolished cell apoptosis induced by ischemia and hypoxia in cardiomyocytes via stimulating signaling tyrosine kinase FGFR2, which was associated with PI3K–AKT signaling pathway. In summary, lncRNA FAF regulated apoptosis through positively controlling FGF9/FGFR2 by influencing the PI3K–AKT signaling pathway [30].

#### Testis-specific transcript Y-linked 15

By analyzing the gene expression profile dataset GSE66360 in peripheral blood samples of MI patients, lncRNA testis-specific transcript Y-linked 15 (TTTY15) was identified to be highly detectable during MI. Similarly, TTTY15 expression was tremendously up-regulated in human cardiomyocyte primary cells (HCMs) under hypoxia condition. TTTY15 knockdown protected HCMs against hypoxia through decreasing cell apoptosis and recovering cell migration and invasion, whereas these effects were counteracted by silencing of miR-455-5p, a target of TTTY15 identified in previous studies. Moreover, TTY15 could regulate the expression of Jun dimerization protein 2 (JDP2), a downstream target of miR-455-5p, by sponging miR-455-5p in hypoxia-treated HCMs. Summarily, the results showed TTTY15–miR-455-5p–JDP2 axis played a key regulatory role in hypoxia-induced injury in HCMs, which provided new evidence for understanding the role of lncRNAs in MI [31].

#### Endogenous cardiac regeneration-associated regulator

Chen et al. [32] screened 1343 up-regulated lncRNAs and 1749 down-regulated lncRNAs were in fetuses of human hearts compared with adult hearts. Among the up-regulated lncRNAs, a novel lncRNA called endogenous cardiac regeneration-associated regulator (ECRAR) was identified to increase rat post-natal cardiomyocytes (CMs) proliferation *in vivo* and *in vitro*. Increased expression of ECRAR enhanced rat post-natal CM proliferation after MI and improved post-MI cardiac function in rats, while silencing of ECRAR showed fewer proliferating post-natal day 1 (P1) CMs and aggravation of cardiac function after MI. Mechanistic analysis revealed that E2F transcription factor 1 (E2F1) was responsible for ECRAR expression in CMs. Moreover, ECRAR activated the phosphorylation of ERKs 1 and 2 (ERK1/2) signaling to trigger post-natal myocardial regeneration. These results disclose that lncRNA ECRAR may be a novel therapeutic target in HF.

#### Cardiomyocyte proliferation regulator

Ponnusamy et al. [33] performed initial screening and used bioinformatics analysis in the heart from embryo to adult to identify the lncRNAs participating in the regulation of adult cardiomyocyte proliferation. Among the four highly expressed lncRNAs (AK080084, AK008146, AK138537, and AK006531) observed in adult mice hearts, cardiomyocyte proliferation regulator (CPR) (AK080084) was the only lncRNA that showed to serve as a modulator of adult cardiomyocyte proliferation. Transgenic mice with cardiac-specific expression of CPR (CPR Tg) and CPR knockout (CPR KO) mice were generated to determine the influence of CPR in regenerating heart. Overexpressed CPR attenuated cardiomyocytes regeneration and cardiac function of the neonatal heart after MI. which was in line with the results verified in adeno-associated virus serotype 9 (AAV9)-CPR injected mice heart. In addition, ablation of CPR increased cardiomyocyte proliferation and repaired cardiac function after MI. Next, they investigated the underlying mechanism by which CPR regulates cardiomyocyte proliferation. Minichromosome maintenance 3 (MCM3), a key initiator eukaryotic genome replication, was found to be distinctly increased in the CPR KO mice hearts. Further studies indicated that CPR inhibited cardiomyocytes proliferation in cardiomyocytes and in mice heart through recruiting *de novo* DNA methyltransferases 3A (DNMT3A) which contributes to the DNA methylation, to the promoter CpG sites of MCM3 and silenced its expression [33].

#### LncRNA NR_045363

Wang et al. [34] have previously determined a profile of lncRNAs differentially expressed between the human fetal and adult hearts. Recently, one human embryo-enriched lncRNA antisense to human CDK6 (LOC101927497) has particularly attracted their attention. Using UCSC blat tool, NR_045363, a mice homolog of human LOC101927497 was identified. The authors found that NR_045363 was mostly expressed in cardiomyocytes and hardly expressed in non-cardiomyocytes of mice hearts. NR_045363 overexpression facilitated cardiomyocyte proliferation and ameliorated cardiac function in 7-day-old mice heart after MI. NR_045363 deficiency blocked proliferation of primary embryonic cardiomyocytes. Bioinformatics analysis and luciferase reporter assay revealed that miR-216a was a target of NR_045363. They further demonstrated that miR-216a could potentially bind to Janus kinase 2 (JAK2), a member of the Janus kinase family. Collectively, the data suggested that NR_045363 played an important role in promoting cardiomyocyte proliferation through interaction with miR-216a, which, in turn, activated the JAK2–STAT3 pathway [35].

#### Others

LncRNA Taurine up-regulated gene 1 (TUG1) was first identified in a screen for genes up-regulated in response to taurine in developing retinal cells [36]. Wu et al. [37] have explored the roles of TUG1 in cardiomyocyte injury induced by hypoxia. The expression of TUG1 was significantly higher under hypoxic conditions, and overexpression of TUG1 enhanced hypoxia-induced cell injury via negative regulation of miR-1455p. This phenomenon may represent a key mechanism by which TUG1 regulates hypoxia-induced cardiac injury. Maternally expressed gene 3 (Meg3) has been identified as a maternally expressed imprinted gene located in the distal part of mouse chromosome 12, and the human homolog, the *MEG3* gene, has been found on chromosomal arm 14q [38]. Gong et al. [39] have reported Meg3 expression increased remarkably in rat cardiomyocyte-derived H9c2 cells under hypoxic conditions (12 h). A knockdown of MEG3 was shown to alleviate hypoxia-induced injury in H9c2 cells by regulating miR-183 and its target molecule, p27, via activation of PI3K–AKT–forkhead box class O 3a (FOXO3a) signaling pathway [39].

### The roles of lncRNAs in MI/R injury

Culprit vessel reperfusion therapeutics to rescue myocardium can cause continuous damage to ischemic cardiomyocytes followed by adverse consequences such as MI/R injury. LncRNAs are emerging as key players in MI/R injury (Table 3).

**Table 3:** The roles of lncRNAs in MI/R injury

LncRNA	Models	Expression	Role
RMRP	MI/R in mice and H9c2 cells under hypoxia condition	Up	Knockdown of RMRP could improve cardiac function and reduce the cell apoptosis in mice hearts exposed to MI/R injury [44]
H19	MI/R in mice and neonatal rat primary cardiomyocytes under OGD/R treatment	Up	Knockdown of H19 could improve cardiac function and decrease infarct size of mice hearts after MI/R [49]
UCA1	CIR in rat	Down	Improving cell viability of cardiomyocytes [51]
	H9c2 cells under H/R treatment	Up	Attenuating H/R-induced injury in cardiomyocytes [52]
FTX	MI/R in mice	Down	Inhibiting H_2_O_2_-induced apoptosis in cardiomyocytes [56]
AK088388	HL-1 cells under H/R treatment	Up	Promoting autophagy of cardiomyocytes in HL-1 cells under H/R treatment [57]
HIF1A-AS1	MI/R in mice	Up	Attenuating cells injury and improving cardiac function in mice during MI/R injury [59]

#### RNA component of mitochondrial RNA processing endoribonuclease

LncRNA RNA processing endoribonuclease (RMRP) was a differentially expressed lncRNA originally identified in gastric cancer [40]. The results indicated that RMRP was highly expressed in H9c2 cells under hypoxia condition and overexpression of RMRP provoked hypoxia-induced injury in H9c2 cells via down-regulation of miR-206. In addition, the activation of PI3K–AKT–mTOR pathway induced by RMRP overexpression could be reversed by miR-206 overexpression in H9c2 cells injury in response to hypoxia. Furthermore, miR-206 improved hypoxia triggered cell damage through regulating ATG3, which was predicted and verified as a target of miR-206. In a rat model of MI/R (60  min ischemia and 24  h reperfusion), RMPR inhibition exerts a protective effect against MI/R injury. The findings uncover that RMRP may be a potential target for new strategies for treating MI/R injury [41].

#### H19

H19 is known to be remarkably expressed during fetal development [42]. Some studies have revealed its effects in pathological CH [43], diabetic cardiomyopathy [44], and DCM [45]. Luo et al. [46] recently investigated the mechanism of action of H19 and H19-derived miR-675 in the pathogenesis of MI/R injury. The expression levels of H19 and miR-675 were up-regulated in cardiomyocytes treated with oxygen-glucose deprivation and reperfusion (OGD/R). Knockdown of H19 abated oxidative stress and inflammation through the regulation of the Nrf2–HO-1 signaling pathway and NF-κB signaling pathway, whereas these effects were partly reversed by enhanced expression of miR-675. Moreover, PPARα, a key player in MI/R injury, was validated as a target of miR-675 and could regulate the effects of the H19–miR-675 axis in OGD/R-treated cardiomyocytes. *In vivo* studies further confirmed that H19 ablation remarkably ameliorated cardiac function and decreased infarct size of mice hearts after MI/R. In summary, these findings suggest that H19 could mediate MI/R injury partially due to the regulation of the miR-675–PPARα axis.

#### Urothelial carcinoma-associated 1

lncRNA urothelial carcinoma-associated 1 (UCA1) was first identified as an ncRNA dramatically up-regulated in bladder transitional cell carcinoma [47]. Liu et al. [48] reported that UCA1 is down-regulated upon focal cardiac ischemia–reperfusion injury (30-min myocardial ischemia followed by 24-h reperfusion) in rats. Similar results were obtained in cardiomyocytes subjected to H_2_O_2_ treatment (200 μM). UCA1 expression was inversely related to H_2_O_2_-induced cardiomyocyte apoptosis. Up-regulation of UCA1 enhanced cell viability at 48 and 72 h after transfection, whereas down-regulation of UCA1 decreased cell viability. Moreover, a knockdown of UCA1 increased the number of apoptotic cells in primary cardiomyocyte cultures. The proapoptotic action of UCA1 in primary cardiomyocytes was found to be mediated via down-regulation of p27, a molecule that leads to the activation of cleaved-caspase 3, thereby inducing cell apoptosis after MI/R injury. It was recently reported that UCA1 expression was significantly up-regulated after hypoxia–reperfusion (H/R) treatment in rat H9c2 cells. LncRNA UCA1 could attenuate cell apoptosis, endoplasmic reticulum (ER) stress and mitochondria dysfunction in H/R-treated H9c2 cells, which suggested that UCA1 may be a crucial target against I/R injury [49].

#### Five prime to Xist

LncRNA Five prime to Xist (FTX) is highly conserved and is encoded within the X-inactivation center [50]. It is known to play a part in cancer progression by modulating miRNAs [51,52]. Recently, Long et al. [53] investigated the regulatory function of FTX in MI/R injury of mice and H_2_O_2_-induced apoptosis of cardiomyocytes. In mice, the level of FTX was significantly down-regulated upon MI/R injury (30, 60, and 120 min post-reperfusion). Consistent results were obtained in isolated cardiomyocytes treated with H_2_O_2_ (100 µM). Enhanced expression of FTX was found to inhibit H_2_O_2_-induced apoptosis in cardiomyocytes and to function as an endogenous sponge for miR-29b-1-5p, which may regulate the expression of B-cell lymphoma 2-like protein 2 (BCL2L2), an antiapoptotic protein. A novel model of apoptosis regulation consisting of FTX, miR-29b-1-5p, and BCL2L2 represents a new approach to tackling cardiomyocyte apoptosis related to heart diseases.

#### Others

Wang et al. [54] recently explored the relationship between lncRNA and autophagy in MI/R injury. The expressions of AK088388 were significantly expressed in HL-1 cells under H/R treatment. Bioinformatics analysis and dual-luciferase reporter assay indicated that miR-30a had binding sites on AK088388 and Beclin-1, a key autophagy-related protein. Enforced expression of miR-30a decreased AK088388 and Beclin-1 expression levels in H/R cardiomyocytes. In addition, further results demonstrated that AK088388 regulated autophagy and enhanced cardiomyocyte damage possibly through the miR-30a–Beclin-1 pathway. It was reported that lncRNA hypoxia-inducible factor 1α-antisense RNA 1 (HIF1A-AS1) exerted a regulatory role on varied kinds of human cancers [55]. Xue et al. [56] investigated MI/R induced injury of myocardium to identify the function of HIF1A-AS1 and its molecular mechanisms of action. Previous studies have reported the involvements of miR-204 and suppressor of cytokine signaling 2 (SOCS2) in MI/R injury. This group demonstrated that HIF1A-AS1 silencing and miR-204 up-regulation improved heart function and suppressed cardiomyocytes apoptosis in mice subjected to MI/R injury. Furthermore, HIF1A-AS1 could up-regulate the expression of SOCS2 and down-regulate miR-204 via ceRNA manner. In summary, the data indicated that HIF1A-AS1 mitigated cells injury and ameliorated cardiac function in mice during MI/R injury partly via regulating miR-204–SOCS2 axis.

### The roles of lncRNAs in cardiac remodeling

After exposure to biomechanical stressors or pathological stimuli, the heart can undergo alterations in its structure, mechanisms, and functions [57]. CH, characterized by an increase in cardiomyocyte size and thickening of ventricular walls, is an adaptive response of the heart that is intended to maintain cardiac function as an early response to cardiac overloading [58]. Nonetheless, maladaptive hypertrophy caused by sustained stress is often accompanied by maladaptive cardiac remodeling, leading to an increased risk of HF and sudden death. Therefore, it is essential to discover effective therapeutic targets that suppress maladaptive hypertrophy and consequent HF (Table 4) [59].

**Table 4:** The roles of lncRNAs in cardiac remodeling

LncRNA	Models	Expression	Role
MIAT	Rat cardiomyocytes hypertrophy induced by AngII	Up	Promoting AngII-induced CH in rats [63]
	Mouse CH and NRVMs hypertrophy induced by ISO	Up	Enhancing ISO-induced cardiomyocytes hypertrophy [64]
Chaer	CH in mice induced by TAC	Down	Inhibition of Chaer could alleviate CH induced by TAC [65]
Chast	CH in mice induced by TAC	Up	Chast silencing could attenuate TAC-induced pathological cardiac remodeling [66]
MAGI1-T1	H9c2 cells under AngII treatment	Down	Attenuating AngII-induced cardiomyocyte hypertrophy [68]
CYTOR	CCH in rats induced by AB and AngII-induced cardiomyocyte hypertrophy in H9c2 cells	Up	Knockdown of CYTOR could aggravate cardiomyocyte hypertrophy *in vitro* and *in vivo* [69]
XIST	CH in mice induced by TAC and PE-treated rat cardiomyocytes	Up	Aggravating cardiomyocyte hypertrophy induced by PE [72]
SNHG7	Neonatal rat cardiac myocytes (NRCMs) under AngII treatment	Up	Knockdown of SNHG7 could attenuate AngII-induced cardiomyocyte hypertrophy [73]
Mhrt	CH in mice induced by TAC	Down	Inhibiting CH and failure [76]
UCA1	Mouse CH induced by TAC induced by neonatal cardiomyocytes PE	Up	Promoting the progression of CH [77]
H19	Mouse CH induced by TAC and neonatal ventricular myocytes hypertrophy induced by PE	Up	Reducing cell size both at baseline and in stimulation of PE [46]
SRA1	AAB-induced rat cardiac fibrosis and newborn rat primary cardiac myofibroblasts under AngII treatment	Up	Promoting activation in cardiac myofibroblasts under AngII treatment [79]
MIAT	MI in mice and neonatal mouse cardiac fibroblasts under fetal bovine serum or AngII treatment	Up	Knockdown of MIAT could abate cardiac fibrosis and enhance cardiac function [80]
Meg3	Cardiac remodeling in mice induced by TAC	Up	Inhibition of Meg3 could decrease cardiac fibrosis and ameliorate diastolic dysfunction [81]

Abbreviations: AAB, abdominal aortic banding; Chaer, CH-associated epigenetic regulator; Chast, CH-associated transcript.; PE, phenylephrine; SNHG7, Small nucleolar RNA host gene 7; TAC, transaortic constriction; XIST, X-inactive specific transcript.

#### MI-associated transcript

LncRNA called MI-associated transcript (MIAT), mainly expressed in heart and fetal brain tissues, is associated with cell activities and various diseases. Li et al. [60] have delineated the effect of MIAT in AngII-induced hypertrophic response in rat cardiomyocytes. The expression level of MIAT was detected in cardiomyocytes after treatment with AngII and MIAT expression was found to gradually increase. MIAT was proved to regulate TLR4 expression by sponging miR-93 in cardiomyocytes. The findings confirmed that MIAT ablation could inactivate the PI3K–AKT–mTOR signaling pathway by modulating the miR-93–TLR4 axis against AngII-induced CH. Li et al. [61] have conducted a series of studies to elucidate whether MIAT is involved in the miR-150-5p–P300 pathway. Mice were injected with ISO (20 mg/kg) twice a day, and after 7 days, MIAT expression turned out to be robustly increased in the ISO group compared with control groups. MIAT was also highly expressed in neonatal rat ventricular myocytes treated with ISO. Notably, silencing of MIAT resulted in miR-150 overexpression and down-regulated P300 in neonatal rat ventricular myocytes. The researchers described this MIAT–miR-150-5p–P300 axis as a contributor to the regulation of cardiomyocyte hypertrophy.

#### CH-associated epigenetic regulator

Emerging evidence has highlighted important roles for lncRNAs in epigenetic regulation during heart diseases. Wang et al. [62] have identified nearly 150 lncRNAs that are significantly deregulated in the hypertrophic myocardium in mice after transaortic constriction (TAC) surgery. Expression of a 2737-nt transcript, CH-associated epigenetic regulator (Chaer) was high in cardiac tissue and was found to be necessary for the development of CH. Its expression decreased gradually in failing hearts at 2, 4, and 6 weeks post-TAC. As for the mechanism, Chaer directly binds the catalytic subunit of polycomb repressor complex 2 (PRC2) and suppresses PRC2 function during hypertrophy. This interaction between Chaer and PRC2, which is mediated by a 66-mer motif in Chaer, interferes with targeting of PRC2 to genomic loci thereby leading to inhibition of H3 lysine 27 methylation at the promoter regions of genes. Thus, this interaction is crucial for epigenetic reprogramming and induction of genes taking part in hypertrophy. Moreover, inhibition of Chaer expression in the heart before, but not after, the onset of pressure overload substantially attenuates CH and dysfunction.

#### CH-associated transcript

To identify differentially expressed lncRNAs during pressure overload-induced CH, an lncRNA microarray analysis was performed on mouse hearts with TAC-induced hypertrophy. Among the up-regulated lncRNAs, CH-associated transcript (Chast) was identified as an lncRNA candidate with a role in the pathogenesis of cardiomyocyte hypertrophy. Chast was deregulated between 4 and 13 weeks in failing hearts, with a peak expression level at 6 weeks. GapmeR-mediated silencing of Chast both prevented and attenuated TAC-induced pathological cardiac remodeling without early signs of toxicological side effects. In terms of the mechanism, Chast repressed pleckstrin homology domain-containing protein family M member 1 (PLEKHM1, encoded on the opposite strand relative to the *Chast* gene), thus impeding cardiomyocyte autophagy and driving hypertrophy. These findings indicate that lncRNAs may be potential targets for prevention of cardiac remodeling and highlight a general role of lncRNAs in heart diseases [63].

#### MAGI1 intronic transcript 1

Song et al. [64] identified a highly CH-related lncRNA MAGI1 intronic transcript 1 [MAGI1-IT1] through integrative analysis of a CH-related lncRNA–mRNA network (CHLMN). Zhang et al. [65] unveiled the precise function of MAGI1-IT1 in CH progression. Down-regulation of MAGI1-IT1 was observed in H9c2 cells upon AngII treatment. MAGI1-IT1 overexpression eradicated the enlarged surface area of hypertrophic H9c2 cells and the elevated levels of hypertrophic markers such as atrial natriuretic peptide (ANP), brain natriuretic peptide (BNP), and β-myosin heavy chain (β-MHC). From mechanistic research, it showed that MAGI1-IT1 sponged miR-302e to up-regulate DKK1, a potent inhibitor of the Wnt signaling pathway which is significantly related to CH and was predicted and validated to target miR-302e. Furthermore, MAGI1-IT1 negatively regulated Wnt–β-catenin signaling through modulating DKK1. Taken together, the study indicated that MAGI1-IT1 acted as a promising regulator via inactivating DKK1-mediated Wnt–β-catenin signaling by sponging miR-302e in CH.

#### Cytoskeleton regulator RNA

Yuan et al. [66] performed microarray analysis to screen up-regulated lncRNAs in CH. As is shown in the data from the Genotype-Tissue Expression (GTEx) project, lncRNA cytoskeleton regulator RNA (CYTOR) expression was identified to be highly elevated in CH and cardiomyocyte hypertrophy and was positively correlated with I-κ-B kinase ϵ (IKBKE), which participated in CH through the activation of the AKT and NF-κB signaling pathway. CYTOR knockdown significantly augmented aortic banding (AB)-elicited CH *in vivo* and AngII-induced cardiomyocyte hypertrophy *in vitro*. Bioinformatics analysis and luciferase reporter assay manifested that miR-155, which was in relation to hypertrophic cardiomyopathy (HCM), was a target of both CYTOR and IKBKE. CYTOR obviated miR-155-induced repression of IKBKE via a form of ceRNA. Moreover, suppression of miR-155 partly attenuated the decreased levels of IKKi protein and the activation of NF-kB signaling pathway induced by CYTOR inhibition. Collectively, CYTOR attenuated CH possibly attributed to the regulation of the miR-155–IKKi axis and NF-kB signaling pathway, indicating CYTOR as an effective regulator for CH.

#### X-inactive specific transcript

LncRNA X-inactive specific transcript (XIST) has been widely reported as an oncogene or a tumor suppressor in a wide range of cancers [67]. Recent studies demonstrated the involvement of XIST in MI of mice hearts [68]. XIST has also been reported to be significantly up-regulated in hypertrophic hearts of mice induced by TAC and in phenylephrine (PE)-treated rat cardiomyocytes. Loss- and gain-of-function analyses revealed XIST to be a key contributor to PE-induced cardiomyocyte hypertrophy. Direct binding of XIST to miR-101 in cardiomyocytes has been confirmed by an RIP assay performed in cardiomyocytes with the treatment of PE. Furthermore, Toll-like receptor 2 (TLR2), which is correlated with CH, was verified as a target of miR-101. In conclusion, these findings suggest that XIST may function as a therapeutic target for the treatment of CH through its modulation on the miR-101–TLR2 axis [69].

#### Small nucleolar RNA host gene 7

LncRNA small nucleolar RNA host gene 7 (SNHG7) was identified as an important oncogene and played pre-eminent roles in several cancers [70,71]. Jing et al. [72] uncovered the effects of SNHG7 on CH. The expression of SNHG7, as well as SDA1 domain containing 1 (SDAD1), a novel protein determined in human cancers, was raised in AngII-induced neonatal rat cardiac myocytes (NRCMs) hypertrophy. SNHG7 deficiency improved AngII-induced CH. To investigate the mechanism by which SNHG7 exerted its function, the relationship between SNHG7 and SDAD1 was confirmed. It showed that SNHG7 silencing counteracted the up-regulation of SDAD1 mRNA and protein level in AngII-treated cardiomyocytes. Further studies implied that SNHG7 improved SDAD1 mRNA stability through binding to Hu Antigen R (HuR), a key RNA-binding protein (RBP) largely involved in the regulation of mRNA expression. On the whole, the study indicates that SNHG7 may be a powerful regulator in CH through mediating the stabilization of SDAD1 mRNA.

#### Others

Myosin heavy chain-associated RNA transcript (Mhrt), heart-specific lncRNA abundant in the adult heart, has been identified as a cardioprotective lncRNA by Han et al. [73] Their studies also revealed reduced CH and fibrosis in Mhrt779 transgenic mouse hearts compared with wild-type mouse hearts. Reciprocal Mhrt–Brg1 (the latter is a chromatin-remodeling factor) inhibition constituted a feedback circuit critical for maintaining cardiac function. Zhou et al. [74] have illustrated the biological roles of lncRNA UCA1 in CH. UCA1 was up-regulated in TAC-treated mouse hearts and PE-treated cardiomyocytes. A knockdown of UCA1 abated the increase in cardiomyocyte size and elevated expression of genes *ANP* and *BNP* induced by PE. UCA1 promoted the progression of CH and enhanced the expression of *HOXA9* mRNA through competitive binding with miR-184. LncRNA H19 was also found to participate in cardiomyocyte hypertrophy. H19 and its encoded miR-675, which can inhibit cardiomyocyte hypertrophy, were verified to be up-regulated in pathological CH and HF. H19 overexpression reduced cell size, both at baseline and after treatment with PE, whereas a knockdown of H19-induced cardiomyocyte hypertrophy. Furthermore, *CaMKIIδ* mRNA was identified as a direct target of miR-675 and partially influenced the effect of H19 on cardiomyocyte hypertrophy [43].

It was reported that AngII stimulation, TAC operation or other methods for inducing CH models could also activate cardiac fibroblasts (CFs) in hearts of mice and rats, resulting in excessive extracellular matrix deposition and reduced compliance of heart muscle. Cardiac fibrosis causes substitution of the normal myocardium with a nonfunctional fibrotic tissue, thus leading to systolic and diastolic dysfunction and development of progressive HF [75]. Although it is well recognized that CFs are the main orchestrators and maintainers of an orderly structured extracellular matrix, less is known about their involvement along with lncRNAs in the onset and development of cardiac fibrosis.

LncRNA steroid receptor co-activator 1 (SRA1) is known to exhibit many characteristics as a transcriptional co-activator. Zhang et al. [76] have found that SRA1 expression was significantly increased in abdominal aortic banding (AAB)-induced rat cardiac fibrosis and in newborn rat cardiac myofibroblasts exposed to AngII and that SRA1 overexpression markedly stimulated cardiac myofibroblasts activity. Further, SRA1 promoted cardiac myofibroblast activation by inhibiting expression of miR-148b, which is a direct target of SRA1 in AngII-treated cardiac myofibroblasts. MIAT has been identified as the first cardiac profibrotic lncRNA that may have a role in the pathogenesis of MI. Up-regulation of MIAT was consistently observed in CFs treated with fetal bovine serum (FBS) or AngII. MIAT overexpression induced the dysregulation of miR-24, Furin, and TGF-β1, which are related to cardiac fibrosis. A knockdown of MIAT attenuated cardiac fibrosis and enhanced cardiac function. The authors also confirmed that MIAT may function as a miR-24 sponge in CFs [77]. Piccoli et al. [78] have identified Meg3, which, as mentioned above, is mostly expressed in CFs and transcriptionally down-regulated during late cardiac remodeling. Inhibition of Meg3 decreased cardiac fibrosis and ameliorated diastolic dysfunction *in vivo* after TAC by preventing cardiac MMP2 induction [78].

### Other myocardial pathophysiology

#### Doxorubicin-induced cardiomyopathy

Doxorubicin (DOX) is a useful drug that can effectively kill cancer cells. DOX-induced cardiomyopathy is a cardiotoxic side effect of DOX. Zhang et al. [79] have validated that lncRNA (forkhead box protein C2) FOXC2-AS1 may enhance DOX resistance in osteosarcoma (OS). Lately, the involvement of lncRNA FOXC2-AS1 in DOX-induced cardiotoxicity was investigated. The study showed that the expression levels of FOXC2-AS1 and WNT1-inducible signaling pathway protein-1 (WISP1) mRNA, a factor that could repress the cardiomyocyte death evoked by DOX, were reduced in mice cardiac tissues under DOX treatment than in control group. Moreover, FOXC2-AS1 and WISP1 mRNA expression were positively correlated in DOX-treated mice. FOXC2-AS1 improved the cell viability and up-regulated WISP1 in mice cardiomyocytes with DOX-induced injury. Therefore, it is showed that lncRNA FOXC2-AS1 may be an important cardioprotective factor against DOX-induced cardiotoxicity via elevating WISP1 mRNA expression [80]. Valsartan can modulate the TGF-β1 pathway, and alleviate DOX-induced HF by up-regulating an lncRNA called *CH-related factor* (CHRF) [81]. Obestatin can significantly improve left-ventricle contractility dysfunction caused by DOX. Mhrt is up-regulated in primary cardiomyocytes cotreated with obestatin and DOX, and overexpression of cellular Mhrt by means of pcDNA-Mhrt can up-regulate NRF2 in DOX-incubated cardiomyocytes (Table 5) [82].

**Table 5:** The roles of lncRNAs in other myocardial pathologies

LncRNA	Models	Expression	Role
FOXC2-AS1	DOX-induced cardiotoxicity in mice	Down	Inhibiting DOX-induced cardiomyocyte death [83]
CHRF	HF in mice under DOX treatment	Up	Promoting myocardial cell apoptosis [84]
Mhrt	Rat cardiomyopathy under DOX treatment	Down	Improving cell apoptosis of primary cardiomyocytes [85]
MALAT1	IL-6 induced murine septic cardiomyocytes under LPS treatment	Up	Enhancing TNF-α expression in LPS-treated murine HL-1 cardiomyocytes [86]
NEAT1	Sepsis in mice under LPS treatment	Up	Knockdown of NEAT1 could attenuate myocardial injury induced by LPS in mice [88]
HOTAIR	Sepsis in mice under LPS treatment	Up	Silencing of HOTAIR could preserve cardiac function in sepsis mice [89]
Crnde	DCM in mice	Up	Crnde could ameliorate cardiac fibrosis in DCM mice [92]
MALAT1	DCM in rats	Up	MALAT1 knockdown could significantly reduce cardiomyocyte apoptosis and markedly improve left ventricular systolic and diastolic functions [93]
	DCM in mice and mice cardiomyocytes under high glucose treatment	Up	MALAT1 knockdown could reduce mice cardiomyocyte apoptosis induced by high glucose [94]
H19	DCM in rats	Down	Inhibiting autophagy activation and improve cardiac function [95]

#### Sepsis cardiomyopathy

Sepsis is still a major cause of death in intensive care units across the world. Sepsis cardiomyopathy, or sepsis-induced myocardial cell damage, is a complication of severe sepsis and has a poor prognosis.

LncRNA MALAT1 is up-regulated in lipopolysaccharide (LPS)-treated cardiomyocytes. MALAT1 overexpression can enhance TNF-α up-regulation in LPS-stimulated cells. A MALAT1 knockdown was found to render cells less susceptible to LPS-induced apoptosis and to lower the apoptosis rate in HL-1 cells. Consequently, MALAT1 can enhance TNF-α expression partly by up-regulating serum amyloid antigen 3 (SAA3), an inflammatory ligand that can stimulate IL-6 and TNF-α production in LPS-treated cardiomyocytes [83].

Nuclear paraspeckle assembly transcript 1 (NEAT1) was reported to be a key oncogene implicated in various tumors [84]. Wang et al. [85] illustrated that the expression level of NEAT1 was critically increased in the mouse model of sepsis-induced myocardial injury by intraperitoneal injection of LPS. Further results confirmed that NEAT1 knockdown in LPS-treated mice could significantly mitigate myocardial injury and repress the apoptosis of myocardial cells induced by LPS. Furthermore, NEAT1 small interfering RNA reduced myocardial inflammation in LPS group, evidenced by decreased levels of inflammatory markers (IL-1, IL-6, MCP-1, and TNF-α). Mechanistically, NEAT1 knockdown relieved the up-regulation of TLR2 and p-p65 in heart tissues of LPS group, which suggested that NEAT1 controlled LPS-evoked myocardial injury possibly via activating of TLR2–NF-κB signaling pathway.

The role of HOTAIR in LPS-induced sepsis has also been investigated by Wu et al. [86]. HOTAIR turned out to be up-regulated in cardiomyocytes derived from LPS-induced sepsis in mice and to promote the production of circulating TNF-α by activating NF-κB. Moreover, silencing of HOTAIR preserves cardiac function in mice with LPS-induced sepsis [86].

#### Diabetic cardiomyopathy

Diabetic cardiomyopathy is an important cardiovascular complication that develops in diabetic patients. The diabetic heart is characterized by metabolic disturbances and various morphological changes. Overall, these changes result in extracellular cardiac fibrosis, concentric left ventricular hypertrophy, contractile dysfunction, and DCM, which eventually lead to HF [87].

Graham et al. [88] have demonstrated that the lncRNA colorectal neoplasia differentially expressed (Crnde) may be a diagnostic biomarker specificity for colorectal adenomas and cancers. It was recently found that Crnde was specifically expressed in cardiac tissues of both humans and mice as stated in a web database (LocExpress) and negatively correlated with several marker genes of cardiac fibrosis. Using real-time quantitative PCR (RT-qPCR), researchers further verified that Crnde significantly increased in CFs compared with CMs. Subsequently, a mouse diabetic cardiomyopathy cardiac fibrosis model was established to investigate the effects of Crnde on cardiac fibrosis. The results showed that Crnde expression was remarkably elevated and overexpressed Crnde could reduce cardiac fibrosis and ameliorate cardiac function. Moreover, RIP assay displayed that SMAD family member 3 (Smad3), a potent regulator in myofibroblast differentiation of CFs, transcriptionally activated Crnde through directly targeting Crnde. Importantly, Crnde could impede the transcriptional activity of Smad3 on target genes, thus restraining the marker genes expression of myofibroblast in CFs. In general, the findings propose that Crnde may be a promising anti-cardiac fibrosis target for the treatment of diabetic cardiomyopathy [89].

Zhang et al. [90] have illustrated that MALAT1 is involved in the regulation of left ventricular function during diabetic cardiomyopathy. MALAT1 expression was significantly higher in diabetic rats. A MALAT1 knockdown reduced cardiomyocyte apoptosis and markedly improved left ventricular systolic and diastolic function. Additionally, *in vivo* and *in vitro* studies of mice model of diabetic cardiomyopathy, Malat1 expression was revealed to be considerably increased. Malat1 knockdown reduced cell apoptosis of mice cardiomyocytes under high glucose treatment by sponging miR-181a-5p, which may provide a novel target for diabetic cardiomyopathy [91]. High concentration of glucose was found to reduce H19 expression and increase autophagy in cultured neonatal cardiomyocytes. A knockdown of H19 reduced EZH2 occupancy and H3K27me3 binding in the promoter region of *DIRAS3*. Overexpression of H19 down-regulated DIRAS3 expression, promoted mTOR phosphorylation, and inhibited autophagy activation in cardiomyocytes exposed to high glucose concentration [92].

## LncRNAs and clinical biomarkers of CVDs

Classic protein biomarkers are available and clinically useful only for a small number of diseases [93]. LncRNAs have recently been shown to be novel and promising diagnostic or prognostic biomarkers of various cancers, but few studies have evaluated lncRNAs as biomarkers in the context of CVDs [94]. Recently, more lncRNAs were reported to be associated with MI, HF, and other heart diseases, and therefore, were proposed to be biomarkers of some CVDs [3].

The early diagnosis of AMI is crucial for successful treatment to protect cardiac myocytes and save lives. Vausort et al. [95] have measured expression of five lncRNAs in peripheral-blood cells by quantitative PCR: hypoxia inducible factor 1A antisense RNA 2 (aHIF), cyclin-dependent kinase inhibitor 2B antisense RNA 1 (ANRIL), KCNQ1 overlapping transcript 1 (KCNQ1OT1), MALAT1, and MIAT. Levels of hypoxia inducible factor 1A antisense RNA 2, KCNQ1OT1, and MALAT1 were higher in patients with MI than in healthy volunteers, whereas the levels of ANRIL were lower in patients with MI [95]. In addition, the expression of HOTAIR and cardiac troponin I concentration in AMI patients was measured, and the plasma concentration of HOTAIR was found to serve as a potential diagnostic biomarker of human AMI [21]. Plasma UCA1 concentration is decreased in the early stage of AMI and is increased on day 3 post-AMI. The level of plasma UCA1 did not correlate with hypertension or diabetes in AMI patients. Furthermore, the sensitivity and specificity of testing UCA1 was remarkable for predicting AMI. Nevertheless, it was not better than cardiac troponin I and creatine kinase MB isoenzyme (CK-MB) for the diagnosis of AMI [96]. Likewise, heart-specific lncRNA MHRT is significantly elevated in the blood of AMI patients compared with healthy control subjects. The plasma concentration of MHRT was suggested as a diagnostic biomarker of MI in humans with AMI [97]. Tan et al. [98] elucidated that MIAT was positively correlated with IL-6 and TNF-α in serum samples of patients with coronary atherosclerotic heart disease (CAD). Thus, MIAT may serve as a potential marker for diagnosis and prognosis of the CAD.

LncRNAs have also been increasingly studied as potential biomarkers of HF, which is usually the final manifestation of a CVD and cardiac injury [99]. Greco et al. [15] have measured the expression of deregulated lncRNAs during HF in peripheral blood mononuclear cells from 25 HF patients and 18 healthy individuals. They found that CDKN2B-AS1, HOTAIR, and LOC285194 have a potential as biomarkers of HF [15]. Xuan et al. [100] have quantified lncRNAs in plasma samples collected from a total of 104 HF patients and 109 control healthy participants. They explored the potential of 13 known heart-specific or heart-related lncRNAs to serve as circulating biomarkers of HF and identified NRON and MHRT as possible novel predictive biomarkers of HF [100]. Yu et al. [101] have suggested that chronic HF patients with higher circulating UCA1 levels have a lower survival rate compared with those with a lower circulating level via plasma testing of UCA1 in patients with chronic HF. Survival rates in chronic HF predicted by UCA1 turned out to have a tendency similar to that of BNP, whereas the prognosis by means of UCA1 and BNP showed no significant difference [101]. Microarray analysis and real-time PCR validation showed eight candidate lncRNA biomarkers were preliminary identified in plasma samples collected from ten control subjects and ten chronic HF patients diagnosed with DCM. The second cohort consisted of 64 controls and 64 DCM patients, five of the eight candidate biomarkers were further corroborated. Among them, circulating lncRNA ENST00000507296, which could be determined in human-derived primary cardiomyocyte by lncRNA sequencing (lncRNA-seq), was significantly linked with the heart function. Notably, Kaplan–Meier survival curves presented that low expression of circulating ENST00000507296 was correlated with high event-free survival in patients with DCM. Summarily, circulating lncRNA ENST00000507296 could work as a prognostic biomarker in DCM patients [102].

LncRNAs could serve as biomarkers for other heart diseases. Researchers have discovered that lncRNAs are independent predictors of cardiac diastolic function and remodeling in patients with well-controlled type 2 diabetes. Serum levels of long intergenic ncRNA predicting cardiac remodeling (LIPCAR) in type 2 diabetes patients with abnormal diastolic dysfunction were higher than those in patients with normal diastolic function. Circulating lncRNA called *smooth muscle and endothelial cell-enriched migration/differentiation-associated lncRNA* (SENCR) and MIAT levels were independently associated with the left ventricular mass/left ventricular end-diastolic volume ratio. They are also considered independent predictors of left ventricular remodeling in patients with type 2 diabetes [103]. Janina et al. have quantified seven different mitochondrial and cardiac remodeling-related lncRNAs in patients with hypertrophic nonobstructive cardiomyopathy or hypertrophic obstructive cardiomyopathy (HOCM) and in healthy control individuals. They found that lncRNAs uc004cov.4 and uc022bqu.1 levels were significantly higher in patients with HOCM, but not in patients with hypertrophic nonobstructive cardiomyopathy, and suggested that both can predict HOCM and may serve as helpful biomarkers for clinical studies [104]. Frade et al. [105] have found that MIAT overexpression can serve as a biomarker of chronic Chagas disease–associated cardiomyopathy. Researchers have also investigated the association between plasma ANRIL expression and in-stent restenosis (ISR). They found that the expression of plasma ANRIL was significantly greater in patients with ISR after percutaneous coronary intervention. Furthermore, ANRIL was confirmed as an independent risk factor for the incidence of ISR, suggesting that it may be an optimal prognostic factor for ISR [106].

The studies described above have received ample attention. Nevertheless, the potential of this novel class of transcripts as biomarkers of CVD should be explored further [107].

## Conclusion and perspective

This review presents the current literature regarding the functions of lncRNAs and their mechanisms of action in myocardial pathophysiology caused by various factors as well as preventive and therapeutic approaches against them. Further studies are necessary to improve our understanding of lncRNA involvement in the regulation of gene expression and their molecular mechanisms of action during CVDs. Through the findings reviewed herein, specific therapeutic strategies based on lncRNA pathways could be developed to prevent severe cardiac events [108]. Increased understanding of the mechanisms of lncRNAs’ action will provide useful disease information with high predictive potential and novel therapeutic insights into heart diseases.

In search of new strategies for diagnosis and treatment of myocardial damage, it is essential to find new directions for lncRNA researches. The mechanisms of lncRNAs acting as ceRNAs we mentioned here have provided us a valuable insight into the exploration of pathogenesis of heart diseases. In recent years, researchers also focused on the relationship between lncRNA and epigenetic regulatory mechanisms, including DNA and RNA methylation and demethylation, and demonstrated their involvements in cardiac diseases. In addition, exosomes-derived lncRNAs have become a new approach for rescuing myocardium damaged. A growing body of evidence suggests that energy metabolism is as well closely related to the development of CVDs. The studies of lncRNA implicating in energy metabolism may provide important evidence for the diagnosis and efficiency therapy of myocardial diseases. Besides, lncRNAs have been demonstrated to be impressively involved in cardiomyocytes death including apoptosis and autophagy. Likewise, ferroptosis and pyroptosis or other forms of cell deaths have emerged to play vital roles in heart diseases, which may provide novel insights into exploring the functions of lncRNAs. Moreover, new lncRNAs remain to be discovered for diagnosis, prognosis prediction and treatment of CVDs. However, it is still confronted with many challenges of investigating the lncRNA biology. Animal models do not properly mimic heart diseases in humans, which is also a limitation in discovering effective lncRNAs for clinic application. Choosing the suitable animals and groping effective methods to establish models are of great value to study human diseases. Further, improvements of effective tools to target lncRNAs have become a difficult challenge due to the tissue-specific lncRNAs. In addition, quite a few lncRNAs are poorly conserved, hence it is of great importance to validate the expression levels and effects of lncRNAs both in animals and in humans. Notably, treatment strategies on targeting lncRNA remains difficult to apply to the clinic. The potential for functional lncRNAs as a theraputic strategy against heart diseases has been a great concern for researchers.

Thus, overcoming the challenges of determining cardiac-related lncRNAs and their molecular mechanisms may be significantly beneficial to our further investigations in the diagnosis and treatment of CVDs.

## Abbreviations

AKT: serine-threonine kinase
AMI: acute myocardial infarction
AngII: angiotensin II
ANP: atrial natriuretic peptide
BCL2L2: B-cell lymphoma 2-like protein 2
BNP: brain natriuretic peptide
CAD: coronary atherosclerotic heart disease
CAIF: cardiac autophagy inhibitory factor
CF: cardiac fibroblast
CH: cardiac hypertrophy
Chaer: CH-associated epigenetic regulator
Chast: CH-associated transcript
CM: cardiomyocyte
CPR: cardiomyocyte proliferation regulator
CPR KO: CPR knockout
CVD: cardiovascular disease
DCM: dilated cardiomyopathy
DOX: Doxorubicin
ECRAR: endogenous cardiac regeneration-associated regulator
ERK: extracellular regulated protein kinase
FAF: fibroblast growth factor 9-associated factor
FOXC2: forkhead box protein C2
GAS5: growth arrest-specific transcript 5
HCM: human cardiomyocyte primary cell
HF: heart failure
HIF1A-AS1: hypoxia-inducible factor 1α-antisense RNA 1
HOCM: hypertrophic obstructive cardiomyopathy
HOTAIR: HOX transcript antisense intergenic RNA
H/R: hypoxia–reperfusion
IKBKE: I-κ-B kinase ϵ
ISO: isoproterenol
ISR: in-stent restenosis
JAK2: Janus kinase 2
JDP2: Jun dimerization protein 2
KCNQ1OT1: KCNQ1 overlapping transcript 1
lncRNA: long noncoding RNA
LPS: lipopolysaccharide
MALAT1: metastasis-associated lung adenocarcinoma transcript 1
MCM3: minichromosome maintenance 3
Meg3: maternally expressed gene 3
Mhrt: myosin heavy chain-associated RNA transcript
MI: myocardial infarction
MI/R: myocardial ischemia/reperfusion
ncRNA: noncoding RNA
NEAT1: nuclear paraspeckle assembly transcript 1
OGD/R: oxygen-glucose deprivation and reperfusion
PE: phenylephrine
PI3K: phosphoinositide 3-kinase
PRC2: polycomb repressor complex 2
RIP: RNA-binding protein immunoprecipitation
RMRP: RNA processing endoribonuclease
SDAD1: SDA1 domain containing 1
Smad3: SMAD family member 3
SNHG7: small nucleolar RNA host gene 7
SOCS2: suppressor of cytokine signaling 2
TAC: transaortic constriction
TLR2: Toll-like receptor 2
TTTY15: testis-specific transcript Y-linked 15
TUG1: Taurine up-regulated gene 1
UCA1: urothelial carcinoma-associated 1
ULK1: Unc-51-like autophagy activating kinase 1
WISP1: WNT1-inducible signaling pathway protein-1
XIST: X-inactive specific transcript
ZFAS1: Zinc finger antisense 1
β-MHC: β-myosin heavy chain
